# Code status orders in patients admitted to the intensive care unit with COVID-19: A retrospective cohort study

**DOI:** 10.1016/j.resplu.2022.100219

**Published:** 2022-03-07

**Authors:** Emily E. Moin, Daniel Okin, Sirus J. Jesudasen, Nupur A. Dandawate, Alexander Gavralidis, Leslie L. Chang, Alison S. Witkin, Kathryn A. Hibbert, Aran Kadar, Patrick L. Gordan, Lisa M. Bebell, Peggy S. Lai, George A. Alba

**Affiliations:** a Department of Medicine, Massachusetts General Hospital, Boston, MA, USA; b Division of Pulmonary and Critical Care Medicine, Massachusetts General Hospital, Boston, MA, USA; c Department of Medicine, Salem Hospital, Salem, MA, USA; d Division of Pulmonary, Critical Care and Sleep Medicine, Salem Hospital, Salem, MA, USA; e Division of Pulmonary Medicine and Critical Care, Newton-Wellesley Hospital, Newton, MA, USA; f Division of Infectious Diseases, Medical Practice Evaluation Center and Center for Global Health, Massachusetts General Hospital, Boston, MA, USA

**Keywords:** Code status, Critical care, COVID-19

## Abstract

**Purpose:**

Code status orders impact clinical outcomes as well as patients’ and surrogates’ experiences. This is the first multicenter cohort examining code status orders of ICU patients with COVID-19 reported to date.

**Materials and methods:**

This is a retrospective cohort study including adult patients who tested positive for SARS-CoV-2 and were admitted to the ICU at three hospitals in Massachusetts from March 11, 2020 - May 31, 2020. We examined differences in code status orders at multiple timepoints and performed multivariable regression analysis to identify variables associated with code status at admission.

**Results:**

Among 459 ICU patients with COVID-19, 421 (91.7%) were Full Code at hospital admission. Age and admission from a facility were positively associated with DNR status (adjusted OR 1.10, 95% CI 1.05–1.15, p < 0.001 and adjusted OR 2.68, CI 1.23–5.71, p = 0.011, respectively) while non-English preferred language was negatively associated with DNR status (adjusted OR 0.29, 95% CI 0.10–0.74, p = 0.012). Among 147 patients who died during hospitalization, 95.2% (140) died with DNR code status; most (86.4%) died within two days of final code status change.

**Conclusions:**

The association of non-English preferred language with Full Code status in critically ill COVID-19 patients highlights the importance of medical interpreters in the ICU. Patients who died were transitioned to DNR more than in previous studies, possibly reflecting changes in practice during a novel pandemic.

## Introduction

Patients with coronavirus disease 2019 (COVID-19) are frequently admitted to the intensive care unit (ICU) due to critical illness.1., 2., 3., 4. At the time of admission, code status orders are placed to represent the patient’s wishes regarding receipt of intubation for mechanical ventilation and cardiopulmonary resuscitation (CPR). Although code status orders are limited to a small handful of categories,^5^ they ideally represent the synthesis of the medical team’s best efforts to elicit and apply core values from patients and families, review the medical record for previously stated desires, and provide recommendations.^6^

Code status orders in ICU patients influence objective measures like length of stay and mortality.^7^ Because these metrics are frequently used as ICU outcome measures, it is essential to examine the factors that affect code status orders to accurately interpret findings in clinical research. Illness severity and rapid changes in clinical status in the ICU frequently lead to goals of care conversations that may result in changes in code status. Thus, code status order trajectories may also impact outcomes. In addition to the influence of code status orders on objective measures, there are ample data demonstrating the impact of code status orders on the subjective experiences of patients, surrogates, and members of the medical care team.8., 9., 10., 11.

In 2020, as the first surge of COVID-19 emerged in the United States, many hospitals experienced capacity strain that affected their delivery of care.^12^ These resource limitations especially affected the ICU, where unique expertise, high staff-to-patient ratios, and the need for specialized medical equipment complicate crisis response. Developing goal-concordant code status orders is a time and effort-intensive process that may be particularly vulnerable to capacity strain. Likewise, family members are often key stakeholders in these conversations, but visitation restriction policies enacted during the pandemic may have affected the means of communication between the care team and families. Finally, highly publicized concerns regarding limited medical equipment and the potential need for rationing may have colored perceptions of discussions about code status orders among care team and lay stakeholders alike.^13^

Code status orders have previously been examined in critically ill patients^8^ and ICU patients with specific disease processes like the acute respiratory distress syndrome (ARDS).14., 15. More recent work has examined code status trajectories of critically ill patients with COVID-19 admitted to a single medical center^16^, but this represents the largest and only multicenter cohort examining code status orders of ICU patients with COVID-19 to date.

## Materials and methods

This is a retrospective cohort study of all patients with laboratory-confirmed COVID-19 admitted to a medical or surgical intensive care unit (ICU) at three Mass General Brigham hospitals in the Boston, Massachusetts metropolitan area, between March 11, 2020 and May 31, 2020. Patients were screened for inclusion if an infection control flag for COVID-19 risk or confirmed infection was entered into the electronic medical record and they were age 18 or older at the time of admission. COVID-19 diagnosis was verified with manual chart review and based on positive SARS-CoV-2 polymerase chain reaction test of a nasopharyngeal swab or sputum sample performed by the Massachusetts Department of Public Health, a referral laboratory, or the in-hospital clinical laboratory. The study protocol was approved by the Institutional Review Board at Massachusetts General Hospital (2020P001119).

Differences in code status orders at hospital admission, discharge, and/or death were examined. Study data were collected and managed using the REDCap electronic data capture tool hosted at Mass General Brigham.17., 18. Data were abstracted through August 31, 2020 by physicians (EEM, DO, SJJ, NAD, AG, LLC, ASW, LMB, PSL, GAA). Due to a small number of alternative code status orders in our dataset that nonetheless placed limitations on CPR and/or intubation, these were coded as “Do Not Resuscitate (DNR),” while code status orders which did not limit life-sustaining therapies were coded as “Full Code.” Code status orders that explicitly stated “Comfort Measures Only” were coded as “CMO” and separated from other DNR code status orders in our analysis of clinical trajectories. “CMO” patients were separated from those who were merely DNR in the analysis of clinical trajectories because ICU patients with a code status of “CMO” in the participating institutions often receive interventions such as palliative extubation that significantly affect length of stay. In-hospital mortality was defined as death during the index hospitalization. All patients in our cohort reached the endpoint of either death or hospital discharge during the data collection period. Final code status order prior to death, date of code status order change (if applicable), and location within the hospital at time of death were abstracted manually from the electronic medical record by the study team.

Charlson comorbidity index (CCI), Sepsis-related Organ Failure Assessment (SOFA) score and Simplified Acute Physiology Score (SAPS II) were calculated for all patients at admission using standard formulae.19., 20., 21., 22., 23., 24. Admission partial pressure of oxygen (PaO_2_) to fraction of inspired oxygen (FiO_2_) ratio was calculated using PaO_2_ from the arterial blood gas (ABG) with lowest PaO_2_ / FiO_2_ ratio during the first 24 hours of hospital admission. If patients were not intubated during the first 24 hours of hospital admission, FiO_2_ was estimated according to standard conversions from liters per minute (LPM) to FiO_2_.^25^ Continuous variables are presented as median and interquartile range (IQR) and categorical variables are presented as numbers with percentages. The Mann-Whitney U test was performed for continuous variables and the chi-square test (or Fisher Exact, when appropriate) for categorical variables. Pearson correlation coefficients were calculated for binary and continuous variables.

A multivariable logistic regression analysis was constructed to assess variables associated with code status orders at admission. Variables were selected for inclusion based on a combination of clinical judgment and demonstrated association with code status orders in previously published work.^14^ Variables were excluded *a priori* if they were known to be highly collinear with other model variables, in particular excluding race and ethnicity, known to be collinear with non-English preferred language. The final model included the following covariates: age, sex, non-English preferred language, CCI, location prior to admission, admission SAPS II, and admission partial pressure of oxygen (PaO_2_) to fraction of inspired oxygen (FiO_2_) ratio. Adjusted odds ratios (aOR) and 95% confidence intervals (CI) were estimated. Two-sided p-values less than 0.05 were considered significant. All data analysis was performed using R (version 4.0.3).

## Results

A total of 459 patients were admitted to the ICU due to confirmed COVID-19, of which 421 (91.7%) were Full Code at the time of admission and 38 (8.3%) were DNR (Table 1). The median age was 63 (IQR 51–73) and differed significantly between the Full Code and DNR cohorts (62 [50–72] vs 80.5 [70–87], p < 0.001). DNR patients were more likely to identify as White (81.6% vs 54.2%, p = 0.012), non-Hispanic (86.8% vs 50.4%, p < 0.001), and identify English as their preferred language (78.9% vs 48.5%, p < 0.001). Full Code patients were more likely to be admitted from home rather than another facility or institutional setting (85.0% vs 36.8%, p < 0.001).

**Table 1:** Baseline characteristics stratified by code status at ICU admission.

**Characteristic**	**All (N = 459)**	**Full Code (n = 421) (n = 421)**	**DNR (n = 38)**	**p**
Age (median, IQR)	63 (51–73)	62 (50–72)	80.5 (70–87)	<0.001
Male (%)	295 (64.3)	275 (65.3)	20 (52.6)	0.166
BMI (kg/m^2^)	29.6 (26.2–34.3)	29.7 (26.4–34.2)	28.5 (25.2–34.5)	0.220
Race (%)				0.012
White	259 (56.4)	228 (54.2)	31 (81.6)	
Black	40 (8.7)	38 (9.0)	2 (5.3)	
Other	105 (22.9)	102 (24.2)	3 (7.9)	
Unknown	55 (12.0)	53 (12.6)	2 (5.3)	
Ethnicity (%)				<0.001
Hispanic	182 (39.7)	179 (42.5)	3 (7.9)	
Non-Hispanic	245 (53.4)	212 (50.4)	33 (86.8)	
Unknown	32 (7.0)	30 (7.1)	2 (5.3)	
Preferred language (%)				<0.001
English	234 (51.0)	204 (48.5)	30 (78.9)	
Not English	225 (49.0)	217 (51.5)	8 (21.1)	
Residence before hospitalization (%)				<0.001
Home	372 (81.0)	358 (85.0)	14 (36.8)	
Other facility	84 (18.3)	60 (14.3)	24 (63.2)	
Unknown	3 (0.7)	3 (0.7)	0 (0.0)	
Charlson comorbidity index (median, IQR)	3 (2–5)	3 (1–5)	6 (4.3–7)	<0.001
Past medical history (%)				
Prior pulmonary disease	121 (26.4)	114 (27.1)	7 (18.4)	0.333
Malignancy	58 (12.6)	49 (11.6)	9 (23.7)	0.059
Stroke	26 (5.7)	19 (4.5)	7 (18.4)	0.001
Dementia	40 (8.7)	26 (6.2)	14 (36.8)	<0.001
Hypertension	266 (58.0)	231 (54.9)	35 (92.1)	<0.001
Hyperlipidemia	209 (45.5)	185 (43.9)	24 (63.2)	0.035
Coronary artery disease	61 (13.3)	48 (11.4)	13 (34.2)	<0.001
Chronic kidney disease	99 (21.6)	81 (19.2)	18 (47.4)	<0.001
Diabetes mellitus	206 (44.9)	193 (45.8)	13 (34.2)	0.226
Smoking history (%)				0.099
Never	243 (52.9)	228 (54.2)	15 (39.5)	
Active	23 (5.0)	19 (4.5)	4 (10.5)	
Former	131 (28.5)	116 (27.6)	15 (39.5)	
Unknown	62 (13.5)	58 (13.8)	4 (10.5)	

*Definition of abbreviations:* DNR = do not resuscitate; BMI = body mass index; ICU = intensive care unit.

DNR patients had higher CCI (6 [4.3–7] vs 3 [1–5], p < 0.001) and statistically greater prevalence of pre-existing comorbidities including stroke (p = 0.001), dementia (p < 0.001), hypertension (p < 0.001), hyperlipidemia (p = 0.035), and coronary artery disease (p < 0.001, Table 1).

Compared to Full Code patients, DNR patients had more severe illness based upon SOFA score (6 [4–8] vs 7 [4–13], p = 0.028) and SAPS II (32 [24–41] vs 45 [35–66], p < 0.001). Median ARDS severity as measured by PaO_2_ / FiO_2_ ratio did not differ significantly between groups (163 [111–231] vs. 170 [105–246], p = 0.965). DNR patients were more likely to present with acute kidney injury (p = 0.002), cardiac injury or dysfunction (p = 0.004), and shock (p = 0.032). There were no significant differences detected in level of respiratory support during the first 24 hours of admission, including mechanical ventilation, which was provided to 16 (42.1%) patients with an admission code status of DNR (p = 0.171, Table 2).

**Table 2:** Clinical outcomes and interventions stratified by code status at ICU admission.

**Characteristic**	**All (N = 459)**	**Full Code (n = 421) (n = 421)**	**DNR (n = 38)**	**p**
Parameters at hospital admission (median, IQR)				
SOFA score	6 (4–8)	6 (4–8)	7 (4–13)	0.028
SAPS II	33 (25–42)	32 (24–41)	45 (35–66)	<0.001
PaO_2_ / FiO_2_ ratio (mmHg)	163.4 (111.0–231.1)	163.4 (112.8–231.0)	170.4 (105.0–246.0)	0.965
Complications present at admission (%)				
Hypoxia	387 (84.3)	357 (84.8)	30 (78.9)	0.473
Acute respiratory failure	222 (48.4)	202 (48.0)	20 (52.6)	0.704
Acute kidney injury	127 (27.7)	108 (25.7)	19 (50.0)	0.002
Cardiac injury or dysfunction	93 (20.3)	78 (18.5)	15 (39.5)	0.004
Shock	100 (21.8)	86 (20.4)	14 (36.8)	0.032
Respiratory support during first 24 hours of admission (%)				
None	32 (7.0)	30 (7.1)	2 (5.3)	1.000
Mechanical ventilation	248 (54.0)	232 (55.1)	16 (42.1)	0.171
Non-invasive ventilation (CPAP or BiPAP)	6 (1.3)	6 (1.4)	0 (0.0)	1.000
High flow nasal oxygen	26 (5.7)	21 (5.0)	5 (13.2)	0.085
Supplemental oxygen (LPM ≤ 15)	350 (76.3)	321 (76.2)	29 (76.3)	1.000
Clinical outcomes				
Hospital length of stay (days)	22 (13–36)	23 (14–37)	11 (5–18)	<0.001
ICU length of stay (days)	16 (8–24)	17 (10–25)	5 (1–13)	<0.001
Duration of mechanical ventilation (days)	14 (9–19)	14 (9–19)	11 (5–17)	0.050
In-hospital mortality (%)	147 (32.1)	117 (27.8)	30 (81.1)	<0.001

*Definition of abbreviations:* DNR = do not resuscitate; SOFA = Sepsis-related Organ Failure Assessment; SAPS = Simplified Acute Physiology Score; PaO_2_ / FiO_2_ ratio = partial pressure of oxygen (PaO_2_) to fraction of inspired oxygen (FiO_2_) ratio; CPAP = continuous positive airway pressure; BiPAP = bilevel positive airway pressure; LPM = liters per minute; ICU = intensive care unit.

Hospital and ICU length of stay were longer in patients with Full Code status (23 [14–37] vs 11 [5–18], p < 0.001 and 17 [10–25] vs 5 [1–13], p < 0.001, respectively). In-hospital mortality was lower in Full Code patients (27.8% vs 81.1%, p < 0.001). We did not identify a statistically significant difference in duration of mechanical ventilation between code status groups (Table 2).

In multivariable logistic regression, we found associations between DNR code status and age (aOR 1.10, 95% CI 1.05–1.15, p < 0.001), non-English preferred language (aOR 0.29, 95% CI 0.10–0.74, p = 0.012), being admitted from a setting other than a private home (aOR 2.68, CI 1.23–5.71, p = 0.011), and admission SAPS II (aOR 1.04, 95% CI 1.01–1.07, p = 0.003) (Table 3). PaO_2_ / FiO_2_ ratio was not associated with code status (aOR 1.00, 95% CI 0.99–1.00, p = 0.854).

**Table 3:** Multivariable analysis of predictors of DNR code status at admission.

**Factor**	**aOR**	**95% CI**	**p**
Age	1.10	1.05–1.15	<0.001
Sex, male	0.63	0.28–1.45	0.272
Charlson comorbidity index	1.01	0.82–1.24	0.917
Preferred language, not English	0.29	0.10–0.74	0.012
Residence before hospitalization, not private home	2.68	1.23–5.71	0.011
SAPS II	1.04	1.01–1.07	0.003
PaO_2_ / FiO_2_ ratio	1.00	0.99–1.00	0.854

*Definition of abbreviations:* aOR = adjusted odds ratio; CI = confidence interval; SAPS = Simplified Acute Physiology Score; PaO_2_ / FiO_2_ ratio = partial pressure of oxygen (PaO_2_) to fraction of inspired oxygen (FiO_2_) ratio.

**Fig. 1:**
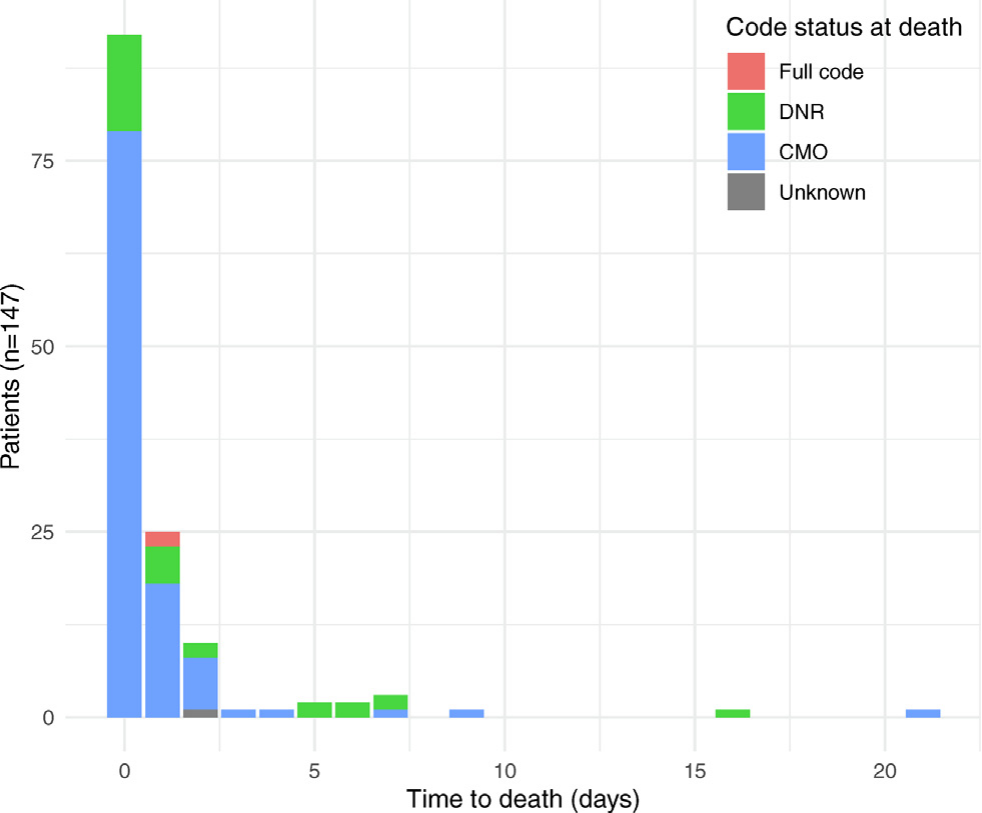
Time to in-hospital death after final code status change. *Legend:* DNR = do not resuscitate; CMO = comfort measures only.

Among the 32.0% (147/459) of patients in our cohort who died while hospitalized, 95.2% (140/147) had a code status order of DNR at the time of death, and 74.8% (110/147) had code status orders indicating CMO. The majority of patients who died were in the ICU at the time of death (131/147, 89.1%) (Table 4), had a change in code status on the same day as their death (92/147, 62.6%), and died within two days of their final code status change (127/147, 86.4%) (Fig. 1). Only eight patients died without experiencing a code status change during hospitalization, of whom four (50%) were DNR at the time of admission.

**Table 4:** Code status orders and patient location at the time of in-hospital death.

**Characteristic**	**In-hospital mortality (n = 147)**
Code status order (%)	
Full code	6 (4.1)
DNR	30 (20.4)
CMO	110 (74.8)
Unknown	1 (0.7)
Location of death (%)	
ICU	131 (89.1)
Non-ICU hospital floor	16 (10.9)

*Definition of abbreviations:* DNR = do not resuscitate; CMO = comfort measures only; ICU = intensive care unit.

## Discussion

In our Massachusetts-based cohort of patients admitted to the ICU with critical illness related to COVID-19, older age, English language preference, White race, and non-Hispanic ethnicity were associated with DNR code status at the time of admission. In multivariable analysis adjusting for potential confounders, only English language preference, higher SAPS II score, and admission from a location other than a private home remained associated with DNR code status at admission.

Although previous work has examined the impact of patient preferred language on goals of care and code status orders in the ICU setting,26., 27. based on a review of the existing literature this is the first analysis to identify non-English preferred language as a predictor of code status orders in critically ill patients with COVID-19. Patient-clinician language discordance is known to impact measures of quality of medical care.28., 29. Discussions of code status and goals of care, when performed well, require nuanced communication that may be hampered when intensive care units operate at or beyond their capacity. When a patient’s respiratory status limits their ability to communicate, as is often the case in patients with respiratory failure due to COVID-19, these challenges are compounded. Although the National Standards for Culturally and Linguistically Appropriate Services in Health and Health Care mandate the provision of language assistance in facilities that receive federal funding,^30^ there is evidence that even prior to the challenges of the COVID-19 pandemic the utilization of professional medical interpreters was inconsistent.^31^ Thus, employing interpreter services may pose a significant enough barrier that clinicians are more likely to have abbreviated conversations and default to a Full Code status.

Likewise, patients and family members may struggle to communicate their wishes when members of the care team do not speak their language. Medical interpreters may have varying degrees of comfort with interpreting sensitive content regarding end of life or limitations to life sustaining therapies,^32^ which may further impair the ability of a language-discordant pair to elucidate the patient or surrogate’s desires. Of note, during the study period the intensive care units included in our analysis employed visitor restriction policies that allowed in-person visitation only “at the end of life” or when patients’ code status orders reflected “comfort measures only.” Thus, many code status conversations with surrogates were conducted by telephone with telephone interpreters on a conference call, rather than in-person, further complicating communication between language-discordant care teams and family members.

Finally, it is possible that preferred language acts as a surrogate for other factors in our analysis. Previous work has described differences in code status that were attributed to cultural differences based on patient race or ethnicity.33., 34., 35., 36., 37. Furthermore, we did not collect data on patient engagement with outpatient medical care prior to hospitalization. Discussions with outpatient providers often lay the groundwork for advance care plans that lead to DNR code status orders in patients admitted to the hospital.^38^ That patients with non-English preferred language also may have fewer and less consistent interactions with providers in the primary care setting^39^ may explain some of the variance in code status orders we identified.

Unlike our study, Mesfin et al.^16^, the only other retrospective cohort examining code status orders in critically ill patients with COVID-19 that we identified in our review of the literature, failed to identify a relationship between code status order and patient preferred language. This may simply reflect a limitation of their smaller sample size; in our study, which was powered to include preferred language in multivariable analysis, the relationship is strong. Differences in practice settings may also explain these findings: their cohort was drawn from a large urban academic medical center that functions as a safety-net hospital and routinely serves a population with significant language diversity. Thus, that center may have had more efficient pre-pandemic workflows in place, further underlining the importance of professional medical interpretation in the ICU setting.

We found that residence in a facility prior to admission was associated with DNR code status at hospital admission in our multivariable model, despite inclusion of likely confounders such as comorbidities and age. Previous studies have examined residence before hospitalization but inconsistently identified a significant relationship with code status.11., 14. Conversely to the explanation above, residence in a facility implies that a patient has engaged to some extent with healthcare providers prior to hospital admission, increasing the likelihood of advance care planning.

Consistent with prior studies in the ICU setting, we identified associations between DNR code status orders and higher CCI,10., 40. as well as markers of acute illness severity such as SOFA and SAPS II scores.15., 41. Unlike prior studies of patients with ARDS, we found no association between PaO_2_ / FiO_2_ ratio and code status at admission in either group comparisons or multivariable analysis.^14^ This may reflect the relative homogeneity of critical illness due to COVID-19 compared to previous ARDS cohorts, where lower PaO_2_ / FiO_2_ ratios may signify more severe overall disease states.^4^ While severity of ARDS as defined by PaO_2_ / FiO_2_ ratio may prompt prognostication that could result in recommendations against Full Code status, for example, lack of experience with a then-novel disease process may have led to discomfort with prognostication that blunted this association. Lastly, it is important to recognize the effect of selection bias in this cohort. Frontline clinicians may have been more aggressive in addressing goals of care and recommending that patients with more severe disease as measured by initial PaO_2_ / FiO_2_ ratio elect for a “do not transfer to the ICU” status. Thus, because our study included only patients admitted to the ICU, those patients are not represented in our analyses.

The vast majority (86.4%) of patients who died in our cohort had a code status change within two days of death. Of patients in our cohort who died, 95.2% had a DNR code status (including “comfort measures only”) at the time of death, which is greater than what has previously been reported in pre-pandemic ICU cohorts.9., 14., 42., 43. Visitation restrictions contingent upon code status orders were in place during the study period; it is possible then that transition to a DNR code status near the end of life was more aggressively pursued when incentivized as a requirement for visitation. Similarly, resource limitations, particularly with regard to adequate personal protective equipment, were of significant concern during the study period.^44^ Early in the pandemic, case series suggested that CPR in patients with COVID-19 was particularly ineffective and placed healthcare workers at significant risk of transmission.^45^ This belief, which has since been contradicted in larger studies,^46^ may have led clinicians to recommend a code status of DNR more often in patients in whom they believed the benefits of CPR to the patient would not outweigh the risks of CPR to the care team.

Our study has several strengths. Our cohort is the largest examining code status orders and trajectories in this population to date, and includes patients admitted to a quaternary referral center ICU as well as multiple community ICUs. Because clinical data were abstracted manually by a team of physicians, the rate of missingness is low. Unlike similar studies that utilized administrative databases to study code status orders, we were able to capture code status orders at multiple timepoints and thus describe code status trajectories during hospitalization. The study period captures the peak of the COVID-19 surge in the Boston area and thus reflects care subject to the greatest magnitude of systems-level shocks, changes in standard processes, and capacity strain.

The study also has notable limitations. Although it includes three medical centers, all were in the same metropolitan region and health system, limiting the generalizability of our findings. As noted above, our analysis included only patients admitted to the ICU, thus excluding patients whose goals of care or code status orders precluded ICU admission. Due to capacity strain, there were extensive emergency department-based palliative care efforts in place in one of the study hospitals,^47^ which likely prevented more ICU admissions and further biased our results. Patient preferred language was abstracted programmatically from the electronic medical record, which is known to contain inaccuracies.^48^ Furthermore, code status orders for critically ill patients were frequently discussed instead with surrogates, complicating the interpretation of our findings. Additionally, it is not necessarily true that non-English preferred language correlates with proficiency in a language other than English. Finally, our study period was short, spanning less than three months during the early days of the pandemic. Tragically, there have been many additional surges and ongoing morbidity and mortality due to severe COVID-19 both in our region and worldwide. Future work should examine the larger sample size that is now available, assess for changes over the course of the pandemic, and compare our results to those of other health systems and locations. Furthermore, future work should explore the association between patient preferred language and code status demonstrated in this population. By better understanding the effect of professional medical interpreters and interpreter modality (e.g. in-person, telephone, or video), we may learn how to better operationalize equitable care for patients with non-English preferred languages.

## Conclusions

The importance of understanding code status orders—both for their ability to impact our understanding of illness trajectories, and for their importance to patients and families—cannot be overstated. The emergence of the COVID-19 pandemic has further complicated this goal by exacerbating capacity strain and changing the makeup of the ICU patient population. In this study, we identified an association of non-English preferred language with Full Code status in critically ill COVID-19 patients. Additionally, patients who died were transitioned to DNR more than in previous studies, possibly reflecting changes in practice during a novel pandemic. Further research is needed to ensure that goal concordant care for all patients does not become a casualty of the COVID-19 pandemic.

### CRediT authorship contribution statement

**Emily E. Moin:** Conceptualization, Methodology, Software, Formal analysis, Investigation, Writing – original draft, Visualization. **Daniel Okin:** Conceptualization, Methodology, Software, Investigation, Writing – review & editing, Project administration, Data curation. **Sirus J. Jesudasen:** Conceptualization, Methodology, Software, Investigation, Writing – review & editing. **Nupur A. Dandawate:** Investigation. **Alexander Gavralidis:** Investigation. **Leslie L. Chang:** Investigation. **Alison S. Witkin:** Investigation, Writing – review & editing. **Kathryn A. Hibbert:** Investigation, Supervision. **Aran Kadar:** Investigation. **Patrick L. Gordan:** Investigation. **Lisa M. Bebell:** Investigation, Methodology, Writing – review & editing. **Peggy S. Lai:** Conceptualization, Methodology, Software, Investigation, Writing – review & editing, Project administration, Data curation, Supervision. **George A. Alba:** Conceptualization, Methodology, Software, Investigation, Writing – review & editing, Project administration, Data curation, Supervision.
